# The effect of sleep quality on learning engagement of junior high school students: the moderating role of mental health

**DOI:** 10.3389/fpsyg.2025.1476840

**Published:** 2025-01-29

**Authors:** Zhenguo Xu, Menghui Niu, Wenxiu Du, Tongtong Dang

**Affiliations:** ^1^School of Communication, Qufu Normal University, Rizhao, China; ^2^Faculty of Education, Qufu Normal University, Qufu, China

**Keywords:** sleep quality, learning engagement, mental health, influencing mechanism, moderating role

## Abstract

**Introduction:**

A good quality of sleep is not only an important guarantee for students’ academic life, but also an important founding condition for their physical and mental health development. The study aims to explore the relationship between sleep quality and students’ learning engagement, and to deeply analyze the moderating role played by mental health.

**Methods:**

A questionnaire survey was conducted among some junior high school students in Rizhao City, Shandong Province for the research.

**Results:**

The results show that: (1) The main effect of sleep quality on learning engagement is significant (*β* = 0.476, *t* = 1.829, *p* < 0.001), specifically, the better the sleep quality, the higher the students’ learning engagement. (2) The influence of sleep quality on learning engagement is moderated by mental health (*β* = –0.850, *t* = -2.652, *p* = 0.006), that is to say, the influence of sleep quality on learning engagement is more significant for students with poor mental health, and mentally healthy students can effectively alleviate the negative impact of sleep problems on their learning engagement.

**Discussion:**

Research shows the significance of good sleep quality and mental health for students’ learning. The research results provide empirical evidence for schools, families, and policy makers to improve students’ academic achievement and mental health.

## 1 Introduction

Learning engagement refers to students’ active participation into various learning activities, their in-depth thinking on study objects, their energetic capability to cope with challenges and setbacks, with positive emotional experiences throughout the learning process. It is a unity of cognitive, behavioral, and emotional engagement (Zhang, 2012). Behavioral engagement refers to the learner’s high degree of commitment to learning activities, which can reflect the learner’s various performance in the learning process, including concentration, hard working, persistence, etc. Cognitive engagement refers to the learner’s high level of cognitive and psychological input in learning processes, especially the utilization of cognitive strategies and meta cognitive strategies, examples in this regard include deep learning, meaning construction, self-monitoring, etc. Emotional engagement refers to the emotional responses manifested by learners in the learning processes, including positive emotions, positive feelings, learning interests, and a sense of belonging etc. Learning engagement is an important indicator to observe students’ learning processes, an important predictor of academic achievement, a prominent factor to measure the quality of education and the development degree of students, a key factor to assess students’ academic success, and a vital factor for promoting students’ success and improving the quality of education (Çali et al., 2024). In short, how to improve students’ learning engagement is a hot topic in the field of education and psychology.

At the same time, sleep problems are extremely common among adolescents, with about 70% of adolescents suffering from sleep deprivation. This is mainly due to academic stress, excessive use of electronic devices (Zhang et al., 2022), irregular schedules, and poor sleep environment. Lack of adequate sleep directly affects adolescents’ ability to learn, leading to distraction, memory loss, and decreased capability in problem-solving, which overall in turn affects academic performance. In addition, long-term sleep deprivation can also seriously deteriorate, the mental health of adolescents, increase the risk of anxiety, depression and other emotional disorders (Kansagra, 2020), eventually affecting emotional stability and social skills. Lack of sleep may also lead to a series of physiological problems such as weight gain and declined level of immunity (Cheng et al., 2016), which further exacerbates the physical and mental health burden of adolescents. Therefore, it is of critical importance to pay attention to and improve the quality of sleep among adolescents.

There have been studies expounding the impact of sleep quality on academic performance and self-efficacy (Aydin and Aydin, 2024), but there has been lesser researches on the relationship between sleep quality and learning engagement. Previous studies have found that both sleep quality and sleep duration can positively predict academic achievement (Jalali et al., 2020). Additionally, a study on adolescents found that maintaining a regular sleep routine is crucial for learning. Those adolescents with irregular sleep patterns and a tendency to stay up late generally yield poorer learning outcomes. In contrast, adolescents who follow the “going to bed early, getting up early” principle tend to have more stable academic performance (Hershner, 2020). Theoretically speaking, how the different dimensions of sleep quality independently or jointly affect the daytime cognitive functions of junior high school students, such as attention keeping, information processing speed, and working memory capacity, can all become key factors affecting learning engagement. Therefore, this study aims to explore the impact of sleep quality on junior high school students’ learning engagement, while also examining how mental health status (including emotional stability, interpersonal relationships, coping mechanisms, and stress levels) moderates the relationship between sleep quality and learning engagement. The research hypothesizes that: Sleep quality significantly predicts junior high school students’ learning engagement (H1), and mental health moderates the relationship between sleep quality and learning engagement (H2).

### 1.1 The relationship between sleep quality and physical and mental health of adolescents

In recent years, sleep quality, as an important topic in the aspect of adolescent health study, has aroused extensive attentions from the academic world. Studies have shown that good sleep quality is essential for physical and mental health of adolescents. Judging from a physiological perspective, adequate and high-quality sleep can help promote growth hormone secretion among adolescents, support physical development, enhance immune system functionality, and reduce the risk of chronic diseases (Dutil et al., 2022). Additionally, endocrine levels can reflect sleep duration and sleep quality, and studies have proven that there is a relationship between blood glucose content and sleep quality, among which long-term sleep deprivation can increase the prevalence of diabetes (Peng et al., 2021). What’s more, the sleep quality index is significantly positively correlated with perceived stress and perceived physiological symptoms, and the sleep quality index significantly negatively predicts stress systolic blood pressure responsiveness, diastolic blood pressure responsiveness, and heart rate responsiveness, that is, the worse the individual’s sleep quality, the more blunted stress cardiovascular responsiveness in the received acute psychosocial stress task (Ni, 2021). Cheng et al. (2016) using stratified cluster sampling, collected data taken during the period of bedtime and data for metabolic syndrome in a one-year follow-up survey. The researchers found that going to bed late was significantly associated with an increased risk of hazard factors for metabolic syndrome in college students, it’s also worth mentioning that changing bedtime routine within 1 year did not reduce the risk level. Guo (2023) based on the survey of 6 types of high school students in Shanghai, it was found that there were differences in sleep quality of high school students with variations in physical activities (*p* < 0.05), and sleep quality of high school students doing dynamic physical activities was better than that of students doing insufficient physical activity.

In terms of mental health, research has found that sleep disorders such as insufficient sleep, having difficulties in falling asleep, or sleep interruptions are closely related to the onset and development of depressive symptoms among adolescents (Roberts et al., 2002). Furthermore, a longitudinal study has shown that adolescents with chronically short sleep duration (less than 8 h per night) are more likely to experience mood instability, anxiety, and behavioral problems, all of which are associated with poorer mental health (Short et al., 2013). A study led by the Norwegian Institute of Public Health discussed the link between adolescents’ use of electronic devices at night and their sleep quality/mental health status. The study revealed that using electronic devices at night reduces adolescents’ sleep time and increases the risk of attention deficit hyperactivity disorder (ADHD), anxiety, and depression (Hysing et al., 2015). Additionally (Feng et al., 2005), through the investigation of sleep quality of a cohort of medical school students and the analysis of its influencing factors, it was discovered that the correlation between sleep quality and anxiety and depression was statistically significant (*p* < 0.0001; Wang and Zhang, 2024) conducted a questionnaire survey in a medical school in Shanxi Province and found out that there was a significant negative correlation between psychological resilience and sleep quality (*p* < 0.01), that is, the higher the psychological resilience, the better the sleep quality. There was a significant positive correlation between stress perception and sleep quality (*p* < 0.01), that is, the higher the degree of stress perception, the worse sleep quality (higher score represents worse sleep quality). In addition, sleep deprivation may lead to decreased efficiency in emotion regulation and an increased risk of behavioral problems and suicidality (Owens and Weiss, 2017). At the same time, social relationships are also impacted, with adolescents with poor sleep quality tending to report lower peer acceptance and higher feelings of loneliness (Gradisar et al., 2013).

Hence, it can be derived that sleep quality has a profound impact on physical and mental health of adolescents, and good sleep habits not only contribute to the wellness of physical health and normal growth and development, but also serve as the basis for maintaining mental health, improving learning efficiency and social skills (Sivertsen et al., 2017). Therefore, families, schools and societies should work together to take measures to improve sleeping environment of adolescents, cultivate good sleep habits, and provide professional intervention and support when necessary to ensure the all-round development of adolescents.

### 1.2 The relationship between sleep quality and learning

Studies have shown that good sleep quality can help improve students’ academic performance (Sivertsen et al., 2017; Liu, 2024). Musshafen et al.’s study explained the relationship between sleep duration and academic achievement among adolescents. Researchers have found that adequate sleep duration (more than 9 h per night) is significantly associated with higher academic achievement, while insufficient sleep is associated with lower grades and poorer academic effectiveness (Musshafen et al., 2021). Apart from that, varied sleep patterns also have an impact on students’ cognitive functionality. Studies have worked out that regular, high-quality sleep helps enhance attention level, working memory, and problem-solving skills, which are essential for effective learning (Lo et al., 2016). Moreover, a study has proven that a short afternoon break can improve afternoon learning efficiency, helping students to focus better, and promote memory consolidation (Spencer, 2021). Another recent study examined the effects of sleep quality on academic motivation and academic performance among high school students. Studies have pointed out that good sleep quality can not only directly improve learning efficiency, but also indirectly enhance learning motivation by reducing depressive emotions and supporting positive interpersonal relationships, thus contributing to the improvement of academic performance (Jalali et al., 2020).

To sum up, sleep quality has a significant impact on learning performance, involving multiple aspects including memory, attention, emotional regulation and others. Adequate and good sleep quality is an important basis for study and life. Poor sleep quality will lead to learners’ lack of concentration, distraction, drowsiness, memory loss and other problems, and in the long run, it is prone to produce learning burnout and affect learning engagement. Thus, for educational institutions and individuals, attaching importance to sleep health, cultivating good sleep habits and optimizing sleep quality are effective ways to improve learning efficiency and academic achievements, and it covers multiple dimensions such as the quality and quantity of sleep and bedtime activities. Future researches could further investigate personalized sleep intervention strategies to promote optimal learning outcome for learners of different ages and needs. On top of this, this study hypothesized that sleep quality significantly predicted the learning engagement of junior high school students (H1).

### 1.3 Regulating role of mental health

Sleep quality and learning engagement are two important factors that affect students’ academic performance and overall well-being. A good night’s sleep not only helps to consolidate the memorization of information, but also directly correlates to the cognitive function and emotional state of the next day, which further affects the motivation and efficiency of learning procedures. However, as a manifestation of individual internal resources, the mechanism of mental health status in regulating the relationship between the aforementioned two factors has rarely been systematically investigated by academic researchers.

From a theoretical perspective, mental health indirectly affects sleep quality and learning engagement by affecting individuals’ emotional regulation, stress management ability and self-efficacy. For example, high levels of mental resilience can reduce the negative effects of stressful events on sleep at the same time, enhancing persistence and effort-making level in the face of learning challenges (Luthar et al., 2000). In addition, mindfulness, as a mental health promotion strategy, has also been identified to improve sleep quality and enhance concentration during study (Black and Slavich, 2016). Plus, a study on college students spotted that students with high levels of psychological stress had poor sleep quality, and thus showcased lower motivation and engagement in learning (Huang et al., 2024)). Qin et al. conducted a survey on the sleep quality of 900 primary school students from grade 3 to grade 6 in Hunan Province, China, and detected that primary school students in Hunan Province had prominent sleep problems, among which the daytime dysfunction caused by sleep problems was the most serious problem. Learning burnout positively predicted poor sleep quality, and mental health played an intermediary role in the relationship between learning burnout and sleep quality (Qin et al., 2022). Another cross-sectional study proved the mediating effect of positive psychological capital (such as hope, optimism and resilience) on improving sleep quality and enhancing learning engagement through questionnaire survey data (Seligman et al., 2005). Concurrently, emotional state may mediate the relationship between sleep quality and academic performance. For instance, learning burnout can lead to decreased sleep quality, and depressed mood plays a mediating role in this process, and this negative cycle can seriously impair learning motivation and outcomes.

**Figure 1:**
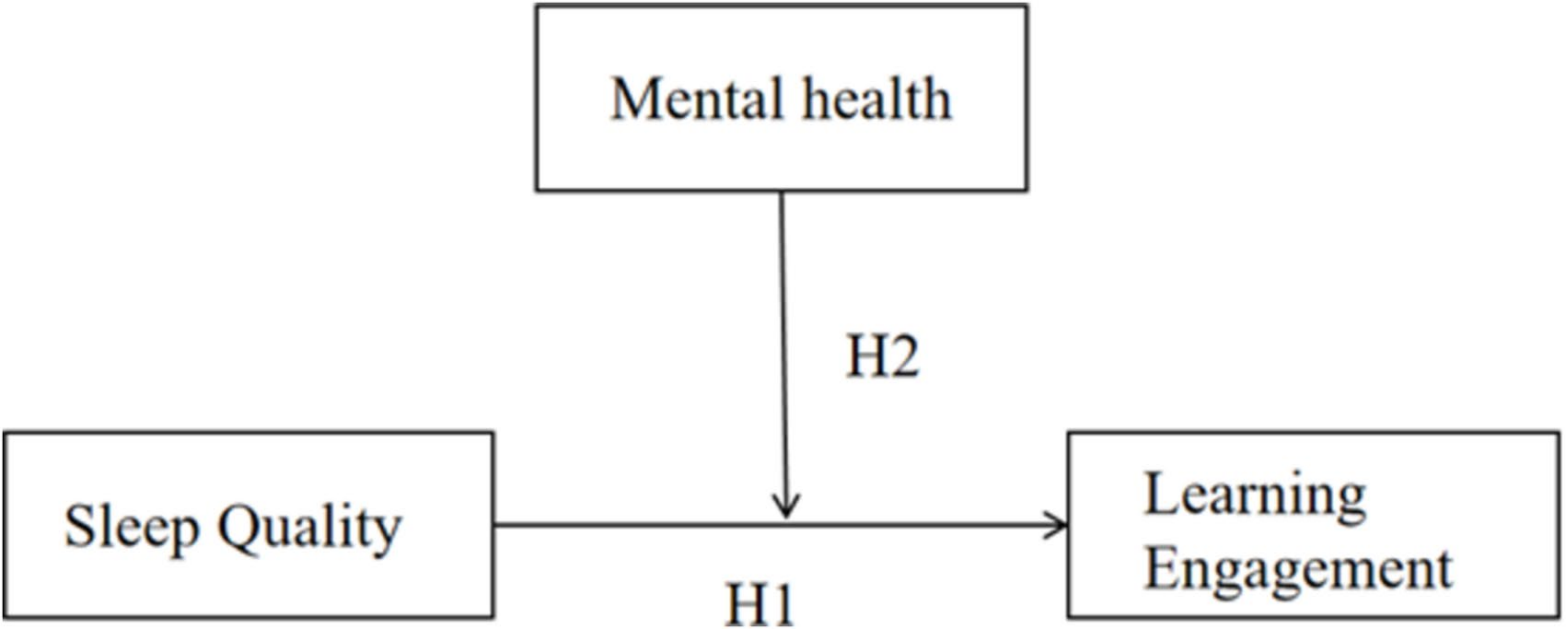
Model diagram.

So, it can be seen that mental health plays a role as a regulator between sleep and learning. On the one hand, mental health problems indirectly reduce learning efficiency by disrupting sleep quality; On the other hand, good mental health can promote positive sleep habits, which can improve learning effectiveness. On these grounds, this study hypothesizes that mental health plays a regulating role in the relationship between sleep quality and learning engagement (H2). The model diagram of this study is shown in Figure 1.

## 2 Methods

### 2.1 Research participants

**Figure 2:**
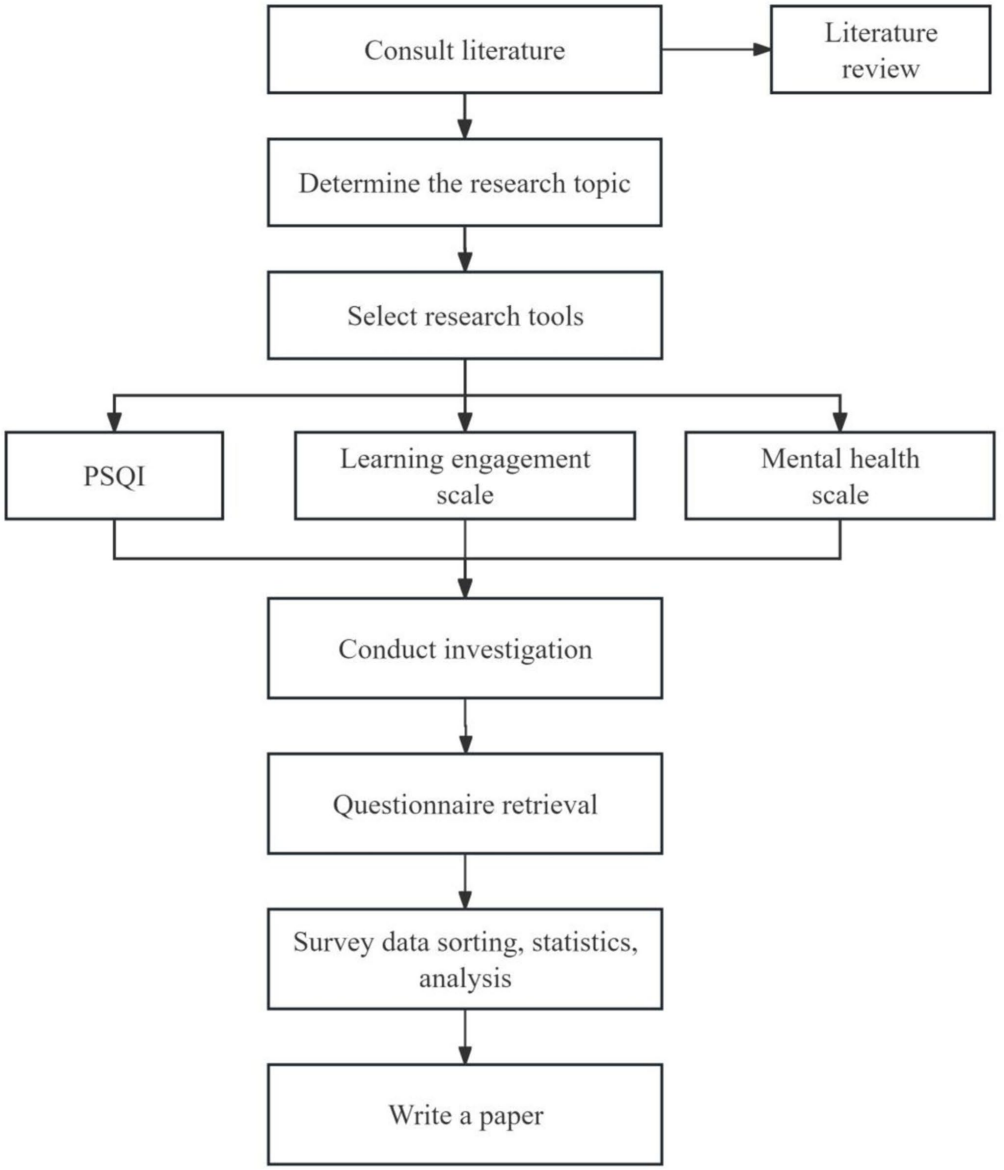
Research roadmap.

In this study, a middle school in Rizhao City, Shandong Province was investigated by using the questionnaire surveying method. The research flow chart of this study is shown in Figure 2. The study had been reviewed and approved by the Ethics Committee of Qufu Normal University (NO. 2024153), and written or oral information had been provided to the subject or his/her legal representative to ensure that the subject or his/her legal representative fully understands the purpose, methods, potential risks and benefits of the study. Researchers had obtained written informed consent forms from subjects or their legal representatives. The research took advantage of the time of students’ self-study class to explain the research intention to students, and emphasized the adopted principles of voluntary, anonymous, and fact-based answer. Additionally, researchers conducted group measurement in class unit. After the participants completed the answer, the questionnaires were recalled on the spot. During the studying period, if the subject has any discomfort, the necessary help will be provided immediately, and the school medical office or hospital will be contacted, and the parents will be informed in time. All data were processed anonymously and would not contain any personally identifiable information. The data is for research purposes only and will not be made public.

To ensure the appropriateness and representativeness of the samples, strict and clear inclusion criteria were set for the respondents. Firstly, from the perspective of school registration and grade, only students with formal junior high school registration and studying in grades one to three were included. Secondly, in terms of the stability of the learning environment, respondents were required not to have frequently transferred schools within the past academic year and to have been studying at the same school. Finally, based on the validity of the research data collection, the included students needed to have basic cognitive, comprehension and expression abilities. A total of 200 questionnaires were distributed, and 193 were returned, with a return rate of 96.5%. After eliminating invalid questionnaires with short answering times and contradictory answers before and after, a total of 186 valid questionnaires were collected, with an effective rate of 96.37%.

The participants’ information in this survey is shown in Table 1. The basic information of the research subjects includes gender, grade, whether they are only children, accommodation or not, and their home location. Among them, there are 89 males and 97 females. There are 66 students in Grade 7, 76 in Grade 8, and 44 in Grade 9. There are 46 only children and 140 non-only children. There are 146 boarding students and 40 non-boarding students. There are 102 urban students and 84 rural students.

**Table 1:** Survey respondents.

Variable	Category	Number	Percentage
Gender	Male	89	47.8%
	Female	97	52.2%
Grade	First	66	35.5%
	Second	76	40.9%
	Third	44	23.6%
Whether you are the only child	Be	46	24.7%
	Not	140	75.3%
Accommodation or not	Be	146	78.5%
	Not	40	21.5%
Home location	Urban	102	54.8%
	Rural	84	45.2%

### 2.2 Research tools

#### 2.2.1 Pittsburgh sleep quality index

The Pittsburgh sleep quality index (PSQI) developed by Buysse, and the domestic researcher (Liu et al., 1996) had translated the revised version. The PSQI has chosen 18 questions (All questions have been included for scoring) to examine 7 dimensions of sleep: sleep quality (睡眠质量), time to fall asleep (入睡时间), sleep duration (睡眠时间), sleep efficiency (睡眠效率), sleep disorders (睡眠障碍), hypnotic drugs (催眠药物), and daytime functionality (日间功能) (Liu et al., 1996). Each dimension is scored 0–3 on a scale of 0–21, with higher scores indicating poorer sleep quality. In this study, Liu Xianchen’s classification standard was used to classify the sleep quality in this study. The overall score is classified as good (0–3), medium (4–7), and poor (8–21). A score of more than 8 is considered to be poor sleep quality and is also identified as having a sleep disorder. All current studies have shown that PSQI has good reliability and validity (Mollayeva et al., 2016), and it maintains good internal consistency. In this study, the retest reliability of PSQI is 0.994, the partial reliability coefficient is 0.824, and the overall Cronbach’s *α* coefficient is 0.912. The fit index χ^2^/df, RMSEA, NNFI, CFI and GFI were 4.83, 0.09, 0.96, 0.98 and 0.97, respectively.

#### 2.2.2 Learning engagement

In this study, the “Learning Engagement Scale for Middle School Students” compiled by Dr. Liu Zaihua and the associate researcher of the Chinese Academy of Education Sciences was used and modified (Wong and Liem, 2022). The scale is divided into three dimensions: behavioral engagement, cognitive engagement, and emotional engagement, and each dimension contains 5 question items. Behavioral engagement includes active learning, teacher-student interaction, group collaboration, etc. Cognitive engagement includes deep learning, meaning construction, and self-monitoring. Emotional engagement includes positive emotions, positive feelings, and learning interest. A five-point scale is used for the scale, with a higher total score representing a higher level of academic engagement. The Cronbach’s *α* coefficient of cognitive engagement was 0.903, the Cronbach’s α coefficient of behavioral engagement was 0.941, the Cronbach’s α coefficient of emotional engagement was 0.940, and the total Cronbach’s *α* coefficient was 0.926. Confirmatory factor analysis of the scale showed that the χ^2^/df was 1.943, the RMSEA was 0.057, and the results of GFI, NFI, IFI, and CFI were 0.849, 0.894, 0.824, and 0.917, respectively, indicating that the structural validity of the learning engagement scale was good, which met the requirements of psychometrics and educational statistics.

#### 2.2.3 Mental health

Chinese Middle School Students Mental Health Scale (MSSMHS) is a mental health scale compiled by Wang Jisheng according to the cultural characteristics and behavior habits of middle school students in China (Wang et al., 1997). It is a symptom-oriented psychological diagnostic scale designed to detect psychological problems or disorders among middle school students. The scale consists of 60 question items, including 10 subscales. They are hostile, anxiety, bigotry, forced, emotional instability, sensitive interpersonal relationships, maladaptation, psychological imbalance, study pressure and depression. A five-point scale is used for the scale, with a higher total score representing worse mental health. The Cronbach’s α coefficient of this scale is 0.953. Confirmatory factor analysis of the scale showed that the χ^2^/df was 1.925, the RMSEA was 0.066, and the results of GFI, NFI, IFI, and CFI were 0.877, 0.826, 0.898, and 0.885, respectively, indicating that the structural validity of the mental health scale was good, which met the requirements of psychometrics and educational statistics.

### 2.3 Data processing

The collected valid data were analyzed using SPSS27.0. First, the HARMAN univariate test was used to detect the presence of common methods bias. Descriptive statistical and correlation analyses were performed on the three variables to determine the relationship between these variables. Finally, hierarchical regression analysis and process macro test were performed to test whether mental health played a moderating role in the relationship between sleep quality and learning engagement.

## 3 Results

### 3.1 Common method bias test and multicollinearity diagnosis

This study utilized a self-report approach, which may exist common methodological biases. As a result, when conducting the questionnaire, the necessary controls were carried out, such as the use of anonymous means to fill out the survey questionnaire, and keeping the confidentiality of the survey results was emphasized, which was used only for this study. According to the suggestions of Podsakoff et al. (2003) and Zhou and Long (2004), the Harman one-way test was carried out, and the analysis results suggested that there were 17 factors with eigenvalues greater than 1 and the variation of the first common factor was 26.11%, and the total variation of the first common factor was below the critical value by 40%. As a consequence, it was considered that there was no obvious common method bias in this study. Also, in order to confirm the existence of multicollinearity between variables, a collinearity diagnosis was performed. The results implied that the variance inflation factor (VIF) values of all items were much smaller than the critical value of 3, so there was no multicollinearity problem.

### 3.2 Descriptive statistical analysis of major variables

#### 3.2.1 Basic information on sleep quality, learning engagement, and mental health of junior high school students

In order to probe into the general situation of junior high school students, the data of sleep quality, learning engagement and mental health were descriptively analyzed.

Table 2 gives out the maximum value, minimum value, average value and standard deviation of sleep quality. The higher the score of sleep quality scale, the worse the sleep quality. The results displayed that the average PSQI score of 186 junior high school students was 5.32 ± 2.19, and the average sleep quality score was 0.96 ± 0.64. The mean sleep duration score was 0.96 ± 0.78. The mean time to fall asleep score was 0.58 ± 0.49. The average sleep efficiency score was 0.09 ± 0.36. The mean sleep disorders score was 0.99 ± 0.37. The average score of hypnotic drugs was 0.05 ± 0.31. The mean daytime functionality score was 1.69 ± 0.88. Based on this result, we can conclude the highest score for daytime functionality and the lowest score for hypnotic drugs. It can be derived that daytime functionality caused by sleep problems is more serious, while there are fewer situations about using hypnotic drugs among junior high school students, which is basically consistent with the results of the study (Qin et al., 2022).

**Table 2:** PSQI scores of junior high school students.

Variable	Maximum	Minimum	Average value	Standard deviation
Sleep quality	3	0	0.96	0.64
Sleep duration	3	0	0.96	0.78
Time to fall asleep	1	0	0.58	0.49
Sleep efficiency	3	0	0.09	0.36
Sleep disorders	2	0	0.99	0.37
Hypnotic drugs	2	0	0.05	0.31
Daytime functionality	3	0	1.69	0.88
PSQI total score	12	0	5.32	2.19

Table 3 refers to the maximum, minimum, average and standard deviation of learning engagement. The higher the score of learning engagement scale, the better the learning engagement. The results indicated that the average score of the 186 junior high school students was 3.51 ± 0.63 in learning and 3.41 ± 0.68 in behavior. The average cognitive engagement score was 3.50 ± 0.72. The average score of emotional engagement was 3.62 ± 0.67. On the basis of this result, we can see that junior high school students have the highest score in the dimension of emotional engagement and the lowest score in the dimension of behavioral engagement.

**Table 3:** Scores of junior high school students’ learning engagement.

Variable	Maximum	Minimum	Average value	Standard deviation
Behavioral engagement	5	1	3.41	0.68
Cognitive engagement	5	1	3.50	0.72
Emotional engagement	5	1	3.62	0.67
Total score for learning engagement	5	1	3.51	0.63

Table 4 shows the maximum, minimum, average and standard deviation of mental health. The higher the score of mental health scale, the worse the mental health status. The results showed that the average mental health score of 186 middle school students was 1.90 ± 0.57, and the hostile factor score was 1.38 ± 0.60. The anxiety factor score was 1.86 ± 0.75. The bigotry factor score was 1.56 ± 0.67. The forced factor score was 2.17 ± 0.75. The emotional instability factor score was 2.37 ± 0.81. The sensitive interpersonal relationships factor score was 1.95 ± 0.78. The maladaptation factor score was 1.92 ± 0.76. The psychological imbalance factor score was 1.51 ± 0.62. The study pressure factor score was 2.61 ± 0.90. The depression factor score was 1.66 ± 0.68. In the light of this result, we can conclude that the study pressure factor score is the highest, the forced and emotional instability factor score is higher, and the hostile, psychological imbalance and bigotry factor score is lower. It draws a conclusion that the problem of learning pressure is more serious in the group of junior high school students. To cope with that, we should pay attention to this issue, jointly arrange students’ study and rest time reasonably, pay attention to students’ emotions, and relieve students’ learning pressure.

**Table 4:** Mental health scores of junior high school students.

Variable	Maximum	Minimum	Average value	Standard deviation
Hostile	4.75	1	1.38	0.60
Anxiety	4.75	1	1.86	0.75
Bigotry	4.25	1	1.56	0.67
Forced	4.50	1	2.17	0.75
Emotional instability	4.75	1	2.37	0.81
Sensitive interpersonal relationships	4.75	1	1.95	0.78
Maladaptation	5.00	1	1.92	0.76
Psychological imbalance	4.25	1	1.51	0.62
Study pressure	5.00	1	2.61	0.90
Depression	4.50	1	1.66	0.68
Total mental health score	4.43	1	1.90	0.57

#### 3.2.2 Gender differences in sleep quality, learning engagement, and mental health of junior high school students

In order to further explore whether there were gender differences in the data, independent sample t test was used to analyze the data. As can be seen from Table 5, *p*-values were all less than 0.05, indicating that there were no significant gender differences in sleep quality, learning engagement and mental health of junior middle school students.

**Table 5:** Gender differences among variables.

Variable	Male (*n* = 89)	Female (*n* = 97)	*t*	*p*	Cohen’s *d*
	M ± SD	M ± SD			
Sleep quality	5.03 ± 2.35	5.57 ± 2.01	−1.682	0.094	0.27
Learning engagement	3.56 ± 0.55	3.47 ± 0.69	1.011	0.313	0.07
Mental health	1.84 ± 0.57	1.95 ± 0.57	−1.391	0.166	0.12

#### 3.2.3 Grade differences in sleep quality, learning engagement, and mental health of junior high school students

In order to further seek whether there were grade differences in the data, analysis of variance was used to analyze the data. In ANOVA, η^2^ is a statistic used to measure the effect size, which represents the proportion of the variability explained by the independent variable to the dependent variable. In other words, η^2^ shows the proportion of the total variation caused by the independent variable. The values of η^2^ range from 0 to 1, with the closer to 1 implying that the independent variable explains more dependent variable variability and therefore the larger the effect; Closer to zero signifies that the independent variable has less influence. As can be viewed from Table 6, there are significant differences in the sleep quality of junior middle school students in grades (*F* = 6.802, *p* = 0.001, η^2^ = 0.49), and the amount of difference is huge. Specifically, the sleep quality of junior third grade is significantly lower than that of junior first grade and junior second grade (the higher the PSQI score, the lower the sleep quality). There was no significant difference in junior middle school students’ learning engagement among grades (*F* = 2.706, *p* = 0.069, η^2^ = 0.29). There were significant differences in the mental health status of middle school students in grades (*F* = 6.504, *p* = 0.0.02, η^2^ = 0.36), and the amount of difference is large, which suggests that the mental health status of the third grade was significantly lower than that of the first and second grades (the higher the score, the worse the mental health status).

**Table 6:** Differences in grade level of each variable.

Variable	First year of junior high school (*n* = 66)	2nd year (*n* = 76)	3rd year (*n* = 44)	*F*	*p*	η^2^
	M ± SD	M ± SD	M ± SD			
Sleep quality	4.76 ± 2.11	5.25 ± 1.96	6.27 ± 2.39	6.802	0.001	0.49
Learning engagement	3.54 ± 0.47	3.59 ± 0.59	3.32 ± 0.84	2.706	0.069	0.29
Mental health	1.73 ± 0.44	1.91 ± 0.61	2.12 ± 0.60	6.504	0.002	0.36

#### 3.2.4 Analysis of sleep quality, learning engagement, and mental health of junior high school students

According to the correlation analysis results in Tables 7–9, the correlations among the variables were as follows: (1) Sleep quality was significantly negatively correlated with cognitive engagement (*r* = −0.157, *p* < 0.05) and emotional engagement (*r* = −0.199, *p* < 0.01); There was a significant negative correlation between sleep duration and emotional engagement (*r* = −0.160, *p* < 0.05). Sleep efficiency was negatively correlated with behavioral engagement (*r* = −0.154, *p* < 0.05) and cognitive engagement (*r* = −0.155, *p* < 0.05). Daytime functionality and behavioral engagement (*r* = 0.214, *p* < 0.01), cognitive (*r* = 0.153, *p* < 0.05) and the emotional engagement (*r* = 0.206, *p* < 0.01) were significantly negative correlation. (2) Sleep efficiency was significantly positively correlated with forced factor (*r* = 0.151, *p* < 0.05), and sleep duration was significantly positively correlated with emotional instability factor (*r* = 0.167, *p* < 0.05) and study pressure factor (*r* = 0.150, *p* < 0.05). (3) Maladaptation was significantly negatively correlated with cognitive engagement (*r* = −0.167, *p* < 0.05) and emotional engagement (*r* = −0.175, *p* < 0.05).

**Table 7:** Correlation between sleep quality and learning engagement.

Variable	Behavioral engagement	Cognitive engagement	Emotional engagement
1. Sleep quality	−0.085	−0.157*	−0.199**
2. Time to fall asleep	−0.05	−0.019	−0.067
3. Sleep duration	−0.089	−0.127	−0.160*
4. Sleep efficiency	−0.154*	−0.155*	−0.066
5. Sleep disorders	−0.084	−0.016	−0.06
6. Hypnotic drugs	0.081	0.044	0.052
7. Daytime functionality	−0.214**	−0.153*	−0.206**

* Represents *p* < 0.05, ** represents *p* < 0.01, and represents *p* < 0.001.

**Table 8:** Correlation analysis between sleep quality and mental health.

Variable	Sleep quality	Time to fall asleep	Sleep duration	Sleep efficiency	Sleep disorders	Hypnotic drugs	Daytime functionality
1. Hostile	0.041	−0.049	0.043	0.003	0.006	−0.002	−0.013
2. Anxiety	−0.031	−0.12	0.003	0.049	0.004	−0.015	−0.095
3. Bigotry	0.027	−0.021	0.061	0.051	0.1	−0.009	0.021
4. Forced	0.022	−0.06	0.124	0.151*	−0.09	−0.039	−0.056
5. Emotional instability	0.048	−0.037	0.167*	0.088	0.013	−0.032	−0.036
6. Sensitive interpersonal relationships	0.061	0.077	0.095	0.038	0.09	0.022	0.04
7. Maladaptation	0.046	0.054	0.041	0.075	0.059	0.013	−0.092
8. Psychological imbalance	0.032	0.012	0.105	−0.034	−0.005	−0.011	−0.053
9. Study pressure	−0.06	−0.093	0.150*	0.091	0.015	−0.045	0.005
10. Depression	0.01	−0.084	−0.016	0.011	0.012	0.011	−0.071

* Represents *p* < 0.05, ** represents *p* < 0.01, and represents *p* < 0.001.

**Table 9:** Correlation analysis between mental health and learning engagement.

Variable	Behavioral engagement	Cognitive engagement	Emotional engagement
1. Hostile	0.011	−0.073	−0.095
2. Anxiety	0.058	−0.02	−0.097
3. Bigotry	−0.1	−0.127	−0.125
4. Forced	0.005	0.025	0.008
5. Emotional instability	−0.008	−0.031	−0.076
6. Sensitive interpersonal relationships	−0.038	−0.051	−0.119
7. Maladaptation	−0.094	−0.167*	−0.175*
8. Psychological imbalance	0.078	−0.004	−0.055
9. Study pressure	−0.052	−0.131	−0.107
10. Depression	0.04	−0.017	−0.088

* Represents *p* < 0.05, ** represents *p* < 0.01, and represents *p* < 0.001.

### 3.3 Mental health moderating test

The results of correlation analysis revealed that sleep quality, mental health and learning engagement were significantly correlated. In order to further probe into the mechanism of sleep quality, mental health and learning engagement among junior middle school students, this study used hierarchical regression analysis to examine whether mental health plays a regulating role in sleep quality and learning engagement. Before the analysis, sleep quality was transformed into a dummy variable. In the regression model, gender and grade were treated as control variables, sleep quality as independent variables, mental health as moderating variables, and learning engagement as dependent variables. A three-step multiple regression analysis was carried out. First, enter gender and grade into the equation as the first control variables. After that, enter sleep quality and mental health into the equation as second-level variables; Finally, enter the product term of sleep quality and mental health into the equation as the third layer variable. In regression analysis, the beta value refers to the standardized regression coefficient. It represents the number of standard deviations of the expected change of the dependent variable (response variable) when the independent variable (predictor variable) changes by one standard deviation. The greater the absolute value of the standardized regression coefficient, the greater the influence of the independent variable on the dependent variable. As can be seen from Table 10, the main effect of sleep quality (*β* = 0.476, *t* = 1.829, *p* < 0.001) and mental health (β = 0.432, *t* = 2.165, *p* = 0.002) was significant, supporting hypothesis H1, that is, sleep quality significantly predicted the learning engagement of middle school students. There was a significant interaction between sleep quality and mental health on learning engagement (*β* = −0.850, *t* = −2.652, *p* = 0.006), supporting hypothesis H2, that mental health plays a moderating role between sleep quality and learning engagement.

**Table 10:** Moderating effect of mental health on the relationship between sleep quality and learning engagement.

Variable	Learning engagement					
	*R* ^2^	∆*R*^2^	*F*(df)	β	*t*	95% CI
Step 1	0.017	0.006	1.578 (2)			
Gender				−0.054	−0.725	[−3.786,1.752]
Grade				−0.109	−1.467	[−3.172,0.467]
Step 2	0.051	0.030	2.435 (4)			
Sleep quality				0.476***	1.829	[0.163,4.272]
mental health				0.432**	2.165	[0.631,13.585]
Step 3 Sleep quality * mental health	0.087	0.061	3.419 (5)	−0.850**	−2.652	[−2.628,-0.386]

* Represents *p* < 0.05, ** represents *p* < 0.01, and represents *p* < 0.001.

**Figure 3:**
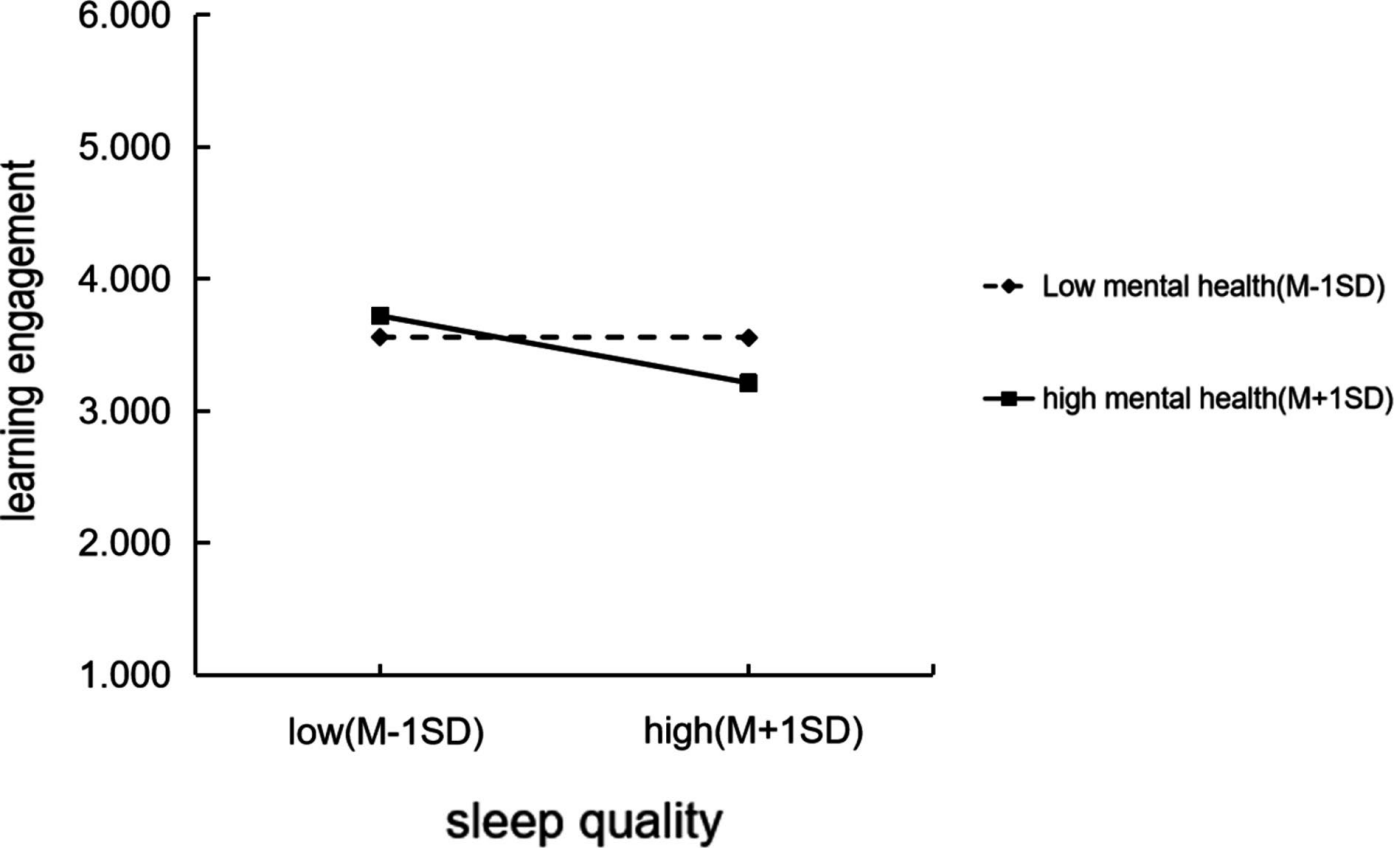
Simple slope plot.

In order to better present the moderating effect of mental health on the relationship between sleep quality and learning engagement, a simple slope analysis was performed by using the point selection method (Fang et al., 2015). Prior to analysis, both mental health and learning engagement were standardized. The predictive effect of sleep quality on learning engagement of junior high school students was calculated by the point selection method when high mental health score (M + 1SD) or high mental health score (M-1SD) were calculated. As can be found from Figure 3, when the mental health score was high (poor mental health status), which was higher than that when the mental health score was low (good mental health status), the negative impact of sleep quality on learning engagement increased (the slope increased), that was, for students with poor mental health, poor sleep quality led to lower learning engagement, while junior high school students with good mental health could better manage emotions, reduce stress, and maintain higher learning enthusiasm and self-efficacy, effective buffering against the negative effects of sleep deprivation even when they had poor sleep quality (higher PSQI scores represent lower sleep quality). The moderating effect of mental health on sleep quality and learning engagement was further verified.

## 4 Discussion

### 4.1 Analysis of the current situation of sleep quality of junior high school students

Pittsburgh sleep quality score > 8 points was used as the reference for adult sleep quality problems in China (Liu et al., 1996). The average score of sleep quality in this study was 5.32, which was less than 8 points, indicating that the overall sleep quality of junior high school students was better. And the maximum value is 12, demonstrating that some students have poor sleep quality. But the actual amount of sleep most middle school students get falls short of the standard recommended by China’s Ministry of Education (General Office of the Ministry of Education, 2021), that is 8 h per day, which is consistent with the founding of Wheaton et al. (2016). Many students go to bed after 11:00 PM, and some students even go to sleep later. This phenomenon is especially obvious when exams approach or they are facing heavy assignment pressure. Students often sacrifice sleep time to complete academic tasks, resulting in insufficient sleep duration, which directly affects their sleep quality. Even if some students are able to get a certain amount of sleep, their sleep qualities are not optimistic. Frequent nighttime awakenings, light sleep, early awakenings and insomnia are common problems. These problems can be caused by academic pressure, using electronic devices late into the night, poor lifestyle habits, or mental health issues (Short et al., 2013). Long-term sleep deprivation will not only lead to the next day’s mental malaise, difficult to concentrate, but also affect memory and learning efficiency, and even cause emotional problems and behavioral disorders (Rey et al., 2020). Besides, the independent sample T-test on sleep quality unveiled that there was no significant difference between the different sexes in terms of sleep quality (*p* > 0.05), which was consistent with the founding of Wheaton et al. (2016). This result may be due to the fact that the group in the study was mainly constituted by residential students, whose life patterns, routines and rest times were very similar, so there was no significant difference overall. There were significant differences in grades (*F* = 6.802, *p* = 0.001, η^2^ = 0.49), and the amount of difference is huge, the specific performance was that the mental health status of the third grade was significantly lower than that of the first and second grades. The possible reason was that the third grade faced the pressure of high school entrance examination, and the learning pressure led to poor sleep state. This is consistent with the study of Talala et al. (2012).

In order to improve the sleep quality of junior high school students, schools, parents and society should make joint efforts. The school can arrange the curriculum reasonably and reduce the unnecessary extracurricular burden; Parents should supervise their children’s use of electronic products to create a more sound sleep environment; In the mean time, students themselves need to develop regular routines and rest habits and learn to effectively manage time and cope with pressure smartly.

In a word, the sleep problem of junior high school students is a public health issue worthy of extensive attention of the society, and requires multi-faceted efforts to solve it in order to ensure the healthy growth of teenagers.

### 4.2 Analysis of the current situation of learning engagement of junior high school students

In this study, the average score of learning engagement was 3.51, and the scores of behavioral engagement, cognitive engagement and emotional engagement were 3.41, 3.50 and 3.62, sequentially. It reflects that most junior high school students have good learning engagement. It can be seen that most junior high school students have a clear cognition of learning, can realize the value and importance of learning, and have a high enthusiasm for learning and a sense of belonging. In the behavioral level, the score of learning engagement is relatively low. Some students actively participate into class activities and take the initiative to ask questions and answer them, while others look more passive and unwilling to participate into class interaction. At the cognitive level, namely in the respects of understanding, analysis and critical thinking, students have varied levels of engagement. Some students are able to think deeply about problems and use critical thinking, while others tend to rote and lack profound understanding and application skills. Although some studies believe that male students have relatively low motivation, concentration and enthusiasm for learning (Wigfield and Eccles, 2000), the results of this study did not find significant gender differences, nor significant grade differences (*p* > 0.05), which may be caused by insufficient sample size and unrepresentativeness.

Family environment, educational resources, teachers’ teaching methods and class psychological environment all affect students’ involvement (Lehrl et al., 2020). A supportive and stimulating environment can stimulate students’ interest and motivation in learning, otherwise it may reduce their engagement level (Meece et al., 2006). Consequently, in order to better promote students’ learning engagement, educators and parents should take measures such as improving teaching methods, enhancing students’ learning interest, providing personalized guidance and support, so as to enhance junior middle school students’ learning engagement and foster their all-round development. At the same time, cultivating students’ self-regulation ability and positive learning attitude is also the key to improve learning engagement.

### 4.3 Analysis of the current situation of mental health of junior high school students

In this study, the average score of mental health is 1.9, proclaiming that most junior high school students have good mental health status, and the maximum value is 4.43, indicating that some students have certain mental health problems, which is basically consistent with most studies (Merikangas et al., 2011). Study pressure is one of the most outstanding manifestations of mental health problems among junior high school students. In the face of fierce competition, especially the approach of the high school entrance examination, many students bear huge academic burdens, long-term high-intensity study not only leads to physical and mental exhaustion, but also may cause anxiety, depression and other emotional disorders. Simultaneously, mood swings are a common phenomenon in adolescence, and some junior high school students lack effective emotional management and self-regulation skills, leading to the accumulation of emotional problems, such as irritability, impulsiveness, depression, etc. Emotional problems not only affect an individual’s academic and interpersonal relationships, but can also evolve into long-term psychological disorders. Furthermore, this study conducted an independent sample T-test on mental health and found that there was no significant difference in gender (*p* > 0.05), but there was a significant difference in grade (*F* = 6.504, *p* = 0.0.02, η^2^ = 0.36), and the extent of difference is large. Specifically, the mental health status of grade 3 was significantly lower than that of grade 1 and grade 2. It can be judged that students in the third grade are under heavy academic pressure and face the pressure coming from study and heavy homework, which not only affects their learning efficiency, but also may lead to anxiety, depression and other emotional problems and more serious mental health problems, which is consistent with the research of Patton et al. (2016).

In order to cope with the challenges of the current mental health of junior high school students, the education system, families and society should work together to provide more comprehensive and professional mental health education and services, including running mental health education courses, establishing psychological counseling mechanisms, and creating a supportive family environment, so as to promote the robust growth of adolescents. Meanwhile, it is necessary to train junior high school students’ self-regulation ability and positive coping strategies to help them build healthy psychological defense mechanisms, which also becomes the key to cope with the current mental health challenges.

### 4.4 Analysis of the current situation of sleep quality of junior high school students

A large number of studies have focused on the relationship between sleep quality and learning engagement, illustrating a profound interaction mechanism between the two. Studies consistently point out that sleep quality is not only the cornerstone of maintaining daily learning effectiveness, but also a key factor in promoting students’ long-term learning engagement and academic achievement (Jalali et al., 2020).

Through hierarchical regression analysis, this study found that sleep quality has a significant main effect on learning engagement (*β* = 0.476, *t* = 1.829, *p* < 0.001), which is consistent with the results of most studies (Dewald et al., 2010), explaining that sleep quality affects the level of learning engagement of junior high school students. The PSQI scale contains 7 dimensions: sleep quality, time to fall asleep, sleep duration, sleep efficiency, sleep disorders, hypnotic drugs and daytime functionality, among which the biggest contribution is daytime functionality (whether there is sleepiness and insufficient energy), with an average score of 1.69 ± 0.88, stating that daytime functionality caused by sleep problems is more serious. Quality sleep is essential for the cognitive function of the brain, and getting enough sleep helps improve attention, memory, decision-making, and creativity, which are all essential elements necessary for learning engagement. Lack of sleep reduces learning efficiency. A tired brain has difficulty in concentrating, slower information processing, and reduced memory, all of which directly affect the learning process. In contrast, a student who gets enough sleep will be able to absorb new information more quickly and review old knowledge more effectively while studying, thus improving learning efficiency. Besides, sleep contributes to emotional stability, reduces anxiety and stress, and enables students to maintain a positive mindset in the face of learning challenges.

### 4.5 Regulating role of mental health

Numerous studies have confirmed that good mental health can cushion the negative effects of sleep deprivation and promote active engagement in the learning process. Students with good mental health are able to demonstrate greater resilience and emotional regulation, as well as maintain higher motivation and engagement in learning, even when faced with occasional sleep disturbances (Lund et al., 2010).

Through hierarchical regression analysis, this study found that sleep quality and mental health have significant interaction effects on learning engagement (*β* = −0.850, *t* = −2.652, *p* = 0.006). Moreover, the simple slope chart shows that when the mental health score is high (poor mental health status), the negative impact of sleep quality on learning engagement increases (the slope increases) compared with that when the mental health score is low (good mental health status), that is, for students with poor mental health status, poor sleep quality will lead to lower learning engagement. However, even in the case of poor sleep quality, junior high school students with good mental health can better manage emotions, reduce pressure, maintain high learning enthusiasm and self-efficacy, and effectively buffer the negative effects of insufficient sleep. When mental health, sleep quality, and learning engagement are in good states, they form a positive feedback loop that feeds on each other and enhances the overall well-being for an individual. This is basically consistent with the study of Windle et al. (2011).

Thus, mental health plays a bridging and regulating role between sleep quality and learning engagement, which not only affects an individual’s sleep pattern, but also determines their attitude and ability to learn. In order to promote the all-round development of students, educators and parents should attach importance to mental health education, provide necessary psychological support and resources, and help students establish healthy living habits and learning strategies to achieve the best sleep quality and learning engagement level. Meanwhile, cultivating positive mental resilience and learning effective stress management and emotional regulation skills are crucial to maintaining good sleep and study status.

## 5 Conclusion

In conclusion, this study explored a model of the moderating effect of mental health on sleep quality and learning engagement. Uncovering the mechanisms behind this relationship could help us understand how sleep quality promotes engagement in learning. The study found that the sleep quality of junior high school students can significantly affect their learning engagement, that is, the better the sleep quality, the higher the learning engagement. A student who gets enough sleep will be able to absorb new information more quickly and review old knowledge more effectively while studying, thus improving learning efficiency. Therefore, schools, parents and society should work together to arrange the curriculum reasonably, reduce unnecessary extracurricular burden, create a good sleep environment for students, so that students can maintain a positive attitude to face the challenges in learning.

The study also found that mental health plays a regulating role between sleep quality and learning engagement, and students with good mental health status can effectively buffer the negative impact of sleep deficiency. In order to promote the all-round development of students, educators and parents should attach importance to mental health education, provide necessary psychological support and resources to help students establish healthy living habits and learning strategies so as to achieve optimal sleep quality and learning engagement. At the same time, cultivating positive mental resilience and learning effective stress management and emotional regulation skills are crucial to maintaining good sleep and study status.

## 6 Limitations and future research

This study also has some shortcomings. For example, all data measurements are based on the perspective of teenagers, which is subjective to a certain extent. In future studies, parents, teachers and students can make objective comments to make up for the shortcomings of self-reported reports. Second, this study only used cross-sectional design to test the mediating mechanism among mental health, sleep quality and learning engagement, and could not draw causal conclusions based on the results. The causal relationship between variables needs to be further investigated and verified in combination with experiments and follow-up studies, so as to further reveal the mechanism of action between variables. Future studies should collect various types of data, such as surveys and interviews with teachers and parents of students, and collect relevant information about junior high school students from one side to improve the reliability of research results. Finally, this study only surveyed junior high school students in one specific province, whose behavioral characteristics and school conditions may differ from those in other regions and other countries, thus limiting the generalizations of the findings. Hence, future studies may consider incorporating the sample of different types of students. At the same time, other factors affecting sleep quality and learning engagement should be further discussed in the future.

## Data Availability

The original contributions presented in the study are included in the article/supplementary material, further inquiries can be directed to the corresponding author/s.
